# Selective autophagy in cancer: mechanisms, therapeutic implications, and future perspectives

**DOI:** 10.1186/s12943-024-01934-y

**Published:** 2024-01-24

**Authors:** Jiaxi Liu, Yongya Wu, Sha Meng, Ping Xu, Shutong Li, Yong Li, Xiuying Hu, Liang Ouyang, Guan Wang

**Affiliations:** 1 Innovation Center of Nursing Research, Nursing Key Laboratory of Sichuan Province, State Key Laboratory of Biotherapy and Cancer Center, National Clinical Research Center for Geriatrics, West China Hospital, Sichuan University /West China School of Nursing, Sichuan University, Chengdu, 610041 China; 2 Emergency Department, Zigong Fourth People’s Hospital, Zigong, 643000 China

**Keywords:** Selective autophagy, Cancer, Molecular mechanism, Small-molecule compounds, Targeted therapy

## Abstract

Eukaryotic cells engage in autophagy, an internal process of self-degradation through lysosomes. Autophagy can be classified as selective or non-selective depending on the way it chooses to degrade substrates. During the process of selective autophagy, damaged and/or redundant organelles like mitochondria, peroxisomes, ribosomes, endoplasmic reticulum (ER), lysosomes, nuclei, proteasomes, and lipid droplets are selectively recycled. Specific cargo is delivered to autophagosomes by specific receptors, isolated and engulfed. Selective autophagy dysfunction is closely linked with cancers, neurodegenerative diseases, metabolic disorders, heart failure, etc. Through reviewing latest research, this review summarized molecular markers and important signaling pathways for selective autophagy, and its significant role in cancers. Moreover, we conducted a comprehensive analysis of small-molecule compounds targeting selective autophagy for their potential application in anti-tumor therapy, elucidating the underlying mechanisms involved. This review aims to supply important scientific references and development directions for the biological mechanisms and drug discovery of anti-tumor targeting selective autophagy in the future.

## Introduction

Autophagy is a process in which intracellular substances like proteins and organelles are transported to lysosomes, degraded and recycled to satisfy the needs of cell metabolism and self-renewal. It not only occurs at the basal level, but is also stimulated by stress and structural restructuring. Autophagy plays a crucial role in the quality control of cytoplasmic components and the maintenance of cellular homeostasis [1]. It has long been believed that autophagy is a nonspecific degradation process. With the progress of autophagy research, it has been found that autophagy can be divided into non-selective autophagy and selective autophagy according to whether there is specificity of substrates to be degraded. Selective autophagy refers to the directed delivery of specific degradation substrates to autophagosomes for degradation by selective autophagy receptors through direct binding to LC3 (Atg8 in yeast and plant cells). At present, the types of selective autophagy include mitophagy, ribosomal autophagy, ER-phagy, pexophagy and so on [2]. Selective autophagy is important for cell differentiation and development, tissue homeostasis, anti-aging, and immunology besides its involvement in cellular homeostasis. It has a close connection to human illnesses like cancer, heart disease, and neurological and infectious diseases [3]. Numerous investigations have revealed that, in the setting of tumor start and growth, selective autophagy contributes to both cell death and survival. In addition to mediating cancer treatment resistance, selective autophagy supplies enough nutrients for cancer and encourages the development, invasion, and metastasis of cancer cells. Nonetheless, it’s demonstrated that selective autophagy can also prevent tumor initiation, development and growth.

Studies have shown that targeting non-selective autophagy has certain risks and limitations in tumor therapy, while targeting selective autophagy may be a more effective strategy for cancer treatment because it can selectively degrade damaged mitochondria, endoplasmic reticulum, and exogenous bacteria and viruses, so as to precisely maintain cell homeostasis. For example, WJ460, a strong inhibitor of myoferritin (MYOF), causes mitophagy in pancreatic ductal adenocarcinoma (PDAC) cells, which results in cell death [4]. Flavagline (FL3) inhibits cancer cell development by mediating Parkin/PTEN-induced kinase 1 (PINK1) dependent mitophagy [5]. Brigatinib triggers the apoptosis of colorectal cancer (CRC) cells via inducing ER stress mediated by oxysterol-binding protein-related protein 8 (ORP8)/ubiquitin-specific peptidase 5 (USP5), and protects ER stress through ER autophagy to optimize cancer treatment [6]. This review primarily summarized signaling pathways and their regulators that are involved in selective autophagy in human cancers, such as the mitophagy receptors autophagy-related gene 32 (Atg32), BCL2/adenovirus E1B 19KDa-interacting protein 3 (BNIP3)/BNIP3-like (NIX), sequestosome 1 (p62/SQSTM1), Parkin/PINK1, NIX, FUN14 domain containing 1 (FUNDC1), and Smad ubiquitin regulatory factor 1 (SMURF1) signaling pathway. The ER-phagy receptors the family with sequence similarity 134 members (FAM134B), reticulon-3 L (RTN3L), SEC62 homolog, preprotein translocation factor (SEC62), cell-cycle progression gene 1 (CCPG1) and Atlastin GTPase 3 (ATL3) and other receptors. At present, most of the literature describes the role of general autophagy in cancer. In this paper, we focus more on the progress of selective autophagy in cancer. We introduced the anti-cancer or carcinogenic autophagy processes of mitophagy, ER-phagy, xenophagy, lipophagy, lysophagy and pexophagy. Finally, we presented a comprehensive analysis of anti-cancer compounds targeting specific selective autophagy pathways and discussed future anti-cancer strategies targeting these autophagic processes based on these findings. The collective insights compiled in this review contribute to the existing scientific knowledge, fostering advancements in the field of selective autophagy-based anti-cancer therapeutics.

## Types of selective autophagy and their mechanisms

Autophagosomes, which are double-membrane spherical vesicles, are formed when the isolation membranes with a small flat membrane structure expand and bend at the start of autophagy. They are carried to lysosomes/vacuoles, where the autophagosome’s outermost membrane fuses with the membranes of vacuoles or lysosomes to generate autolysosomes. The inner membrane of autophagosomes and isolated substances are break down by lysosomes containing hydrolases like lipases, proteases, nucleases and glycosylases. The cytoplasm receives the deteriorated materials and recycles them [7]. Many membrane-associated proteins are involved in the process of autophagy, which happens on membranes [8]. It was not until 2005 that Terje Johansen’s group proposed the process by which autophagy selectively degrades ubiquitinated proteins [9], breaking the long-standing understanding that autophagy is a non-selective degradation process, and the research on selective autophagy is of landmark significance. Differ from the non-selective autophagy, a direct binding between specific receptor proteins and autophagy-related proteins is essential for the transportation of organelles that are damaged or intracellular protein clumps to lysosomes/vacuoles for degradation [10]. LC3-interacting region (LIR), Atg8-interacting motif (AIM) and Atg8 family LC3/GABARAP proteins (Atg8/LC3/GABARAP) are typical features of selective autophagy receptors [11]. Furthermore encouraging autophagosome biogenesis and maturation, Atg8/LC3/GABARAP is also a linker between cargos and the core mechanism of autophagy that ensures the effective recognition and isolation of cargos in autophagosomes. Selective autophagy dysfunction is closely linked with pathological conditions [12, 13]. For example, xenophagy can inhibit tumorigenesis in the early stage, but it also assists tumor cells to cope with microenvironmental pressures like bacterial infection. Mitophagy can either promote or inhibit tumor development in different stages, demonstrating a dual function in immunotherapy, radiation, and chemotherapy as well. Therefore, an in-depth analysis on the molecular mechanisms initiating selective autophagy is of great significance to provide targets and theoretical basis for anti-cancer agents.

### Mitophagy

Mitochondria have a double-membrane structure, which are the primary source of adenosine triphosphate (ATP). Moreover, mitochondria are of great significance in initiating programmed cell death. Mitochondrial dysfunction not only affects cellular homeostasis, but also causes excessive production of reactive oxygen species (ROS) generation and cell death. They are sensitive to the external environment, which can be depolarized and damaged following the stimuli of ROS production, nutrient deficiency and senescence [14]. When mild or moderate damage occurs, to preserve cellular equilibrium, damaged or defective mitochondria are eliminated by the induction of autophagy [15]. When mitochondria are seriously harmed, cytochrome c is released, which starts the apoptotic process. The mitochondrial membrane depolarizes to produce phagocytes with a double-membrane structure that envelops the damaged mitochondria [16]. In *Saccharomyces cerevisiae (S. cerevisiae)*, Atg32 is a protein found in the outer membrane of the mitochondria and is essential for mitophagy. Mammals’ mitophagy, which is mediated by several stress signals and developmental changes, is more complex than that of yeasts [17].

#### Markers of mitophagy

##### Atg32

In *S. cerevisiae*, mitophagy is mainly mediated by Atg32 [18]. When Atg32 is overexpressed, mitophagy is increased, but when it is lost, it almost entirely disappears [17]. The assembly of Atg (autophagy-related gene) proteins is facilitated by Adaptor protein Atg32 connects to Atg11 during mitophagy and serves as a receptor for mitophagy. Among these, there is an interaction between the C-terminal and N-terminal amino acid residues of Atg11 and Atg32. Atg32 is then recruited into the vacuoles alongside mitochondria, where mitochondria are degraded by vacuolar hydrolases [19].

##### BNIP3/nix

BNIP3 and NIX are mitophagy receptors that located on the OMM. They both have an LIR that binds to LC3 directly in order to attract mitochondria to autophagosomes where they are degraded, and they are also contribute to scavenge excess ROS. BNIP3 and NIX have a 56% of homology [20], and both of them has a Bcl-2 homology 3 (BH3) domain for Bcl-2 interaction. Located on mitochondria and ER, BNIP3 and NIX control apoptosis and programmed cell death by influencing mitochondrial respiration and ROS levels. In a hypoxic microenvironment, BNIP3 is essential for an effective mitochondrial turnover [21]. It inserts into the OMM, with the C-terminus and N-terminus in the cytoplasm and mitochondria, respectively. BNIP3 can cause mitochondrial cristae to vanish and promote cytochrome c release. Besides, the phosphorylation of BNIP3 at Ser 17/24 triggers its binding to LC3, thus initiating mitophagy [22].

##### p62

p62/SQSTM1 is the most characterized and earliest discovered autophagy cargo receptor, which reflects the autophagic level [22]. p62 is a multifunctional protein containing an N-terminal Phox-BEM1 domain (PB1), a Z-type zinc finger domain, a nuclear localization signal (NLS), an export motif (NES), a LIR, a keap1 interaction region (KIR), and a C-terminal ubiquitin-associated domain (UBA). p62 assists in preserving cellular homeostasis by triggering the breakdown of protein aggregates and cytoplasts via selective autophagy, contingent upon PB1, LIR, and UBA [23]. p62 establishes a molecular connection between autophagy and ubiquitination. Following the induction of mitophagy, Parkin, an E3 ubiquitin ligase with 465 amino acid residues, recruits p62 to mitochondria. After binding of p62 to ubiquitinated substrates and LC3, ubiquitinated substrates can be incorporated by autophagosomes and destroyed in autolysosomes. p62 also interacts with the 19S proteasome subunit through the PB1 domain, thus providing ubiquitinated substrates for proteasomal degradation. p62 can attach to the proteasome via its ubiquitin (Ub) moiety besides to its UBA domain, which directs the proteasome toward proteasomal destruction [24].

#### Signaling pathways of mediating mitophagy

Parkin-dependent and Parkin-independent mitophagy are the two major mechanisms mediating mitophagy in mammals. The former is mainly referred to Parkin/PINK1-mediated mitophagy, and the latter includes mitophagy mediated by FUNDC1, BNIP3/NIX and SMURF1.

##### Parkin/PINK1 signaling

**Fig. 1:**
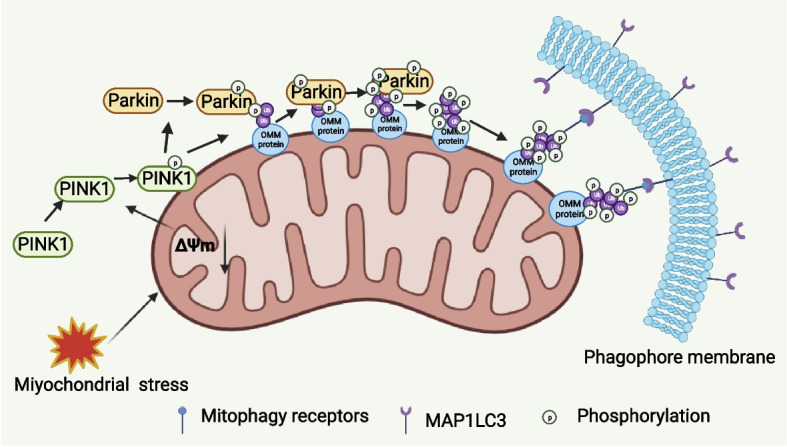
PINK1/Parkin-induced mitophagy. As the mitochondria depolarize, PINK1 steadies and moves to the outer membrane of the mitochondria, where it attracts and phosphorylates parkin. This phosphorylation of ubiquitin molecules causes Parkin to become activated enzymatically. Numerous proteins on the outer membrane of the mitochondria are ubiquitinated by Parkin, which causes autophagy receptors to relocate to the mitochondria where they are selectively recognized by the phagocyte membrane

PINK1 and Parkin are encoded by the Parkinson disease protein 6 (PARK6) and Parkinson disease protein 2 (PARK2) locus, respectively, which are frequently mutant in human cancers. For example, deletion of the PARK2 on chromosome 6q25-q27 is usually detected in a variety of cancers such as breast cancer, bladder cancer [25–27]. PINK1 is a serine/threonine protein kinase located on the OMM, and Parkin is an E3 ubiquitin ligase mainly localized in the cytoplasm [28]. In times of stress, they both control mitochondrial homeostasis. PINK1 is constantly transferred from the OMM to the inner mitochondrial membrane (IMM) in cells with normal and polarized mitochondria, where it is cleaved and broken down. In cases of certain damages like depolarization, the transportation process of PINK1 into the IMM is stagnated. Rather than being cleaved or degraded, PINK1 is accumulated and stabilized on the OMM of damaged mitochondria. Additionally, it phosphorylates Parkin and ubiquitin, and it attracts Parkin to mitochondrial injury. During the recruitment process, Parkin ubiquitinates target proteins on the OMM, and recruits autophagosomes to engulf damaged mitochondria and subsequently degrade mitochondria via lysosomes (Fig. 1) [29].

##### BNIP3/NIX signaling

Hypoxia-inducible factors BNIP3 and NIX both work with LC3-associated phagophores to enhance mitophagy [30]. The N- and C-terminus of the OMM protein BNIP3 are found in the cytoplasm and mitochondria, respectively [31]. BNIP3 is essential for mitophagy at all stages. By blocking mTOR function, BNIP3 prevents the induction of mitophagy during the early stages of the process. Beclin-1 and the BNIP3/NIX BH3 domain later compete with one another for binding to Bcl2 or Bcl-xL, separating Beclin-1 from Bcl2/Bcl-xL. After then, Beclin-1 attaches itself to mitochondria or combines with class III PI3K to initiate mitophagy. During the process of autophagosome maturation [32], BNIP3/NIX promotes the formation of autophagosomes by recruiting LC3 and Gamma-aminobutyric acid receptor-associated protein (GABARAP) [33].

NIX is a receptor for mitophagy in mammals located on the OMM. It is closely related to mitophagy during erythrocyte maturation. During the process of reticulocyte maturation or in a model of mitochondrial damage induced by carbonyl cyanide m-chlorophenyl hydrazone (CCCP), the LIR motif at the N-terminus of NIX can induce mitophagy by recruiting LC3. Notably, mitophagy is markedly inhibited after the removal of the NIX-LC3 domain, suggesting that NIX induces mitophagy depending on the unique autophagy receptor structure [34]. Additionally, NIX and BNIP3 play a role in PINK1/Parkin-mediated mitophagy. Parkin ubiquitinates NIX, which facilitates the selective autophagy adapter neighbor of BRCA1 gene 1 (NBR1) targeting. NBR1 further stimulates the development of autophagosomes surrounding mitochondria by binding to ubiquitin and LC3/GABARAP [35].

##### FUNDC1-mediated mitophagy

FUNDC1, an OMM protein, is a receptor for hypoxia-induced mitophagy [36]. It has an LIR at the N-terminus, and changes to the LIR motif affect how FUNDC1 interacts with LC3 and how mitophagy is induced [37]. The protein level of FUNDC1 is partially regulated by OMM-anchored membrane-associated ring-CH-type finger 5 (MARCH5)/MITOL, which functions as a ubiquitin ligase in the mitochondrial dynamics and ubiquitinates a number of proteins. FUNDC1 is downregulated in a ubiquitin-proteasome-dependent manner under hypoxia as a result of MARCH5-mediated ubiquitination of FUNDC1 at Lys119. Hypoxia-induced mitophagy is enhanced when endogenous MARCH5 is koncked down or when MARCH5 catalytic mutants are overexpressed, as this hinders FUNDC1’s ubiquitination and degradation [38]. Like Atg32 in yeast cells, FUNDC1 is controlled by phosphorylating and dephosphorylating Ser13 and Tyr18, which are situated close to the LIR motif, during mitophagy. Under the normoxic condition, casein kinase 2 (CK2) phosphorylates Ser13, while the serine/tyrosine kinase negatively regulates the FUNDC1-LC3 interaction by mediating the phosphorylation of Tyr18 [37, 39]. Under the hypoxic condition, inactivated serine results in an inhibited phosphorylation of Tyr18, which stabilizes the FUNDC1-LC3 interaction, and promotes mitophagosome formation [37]. PGAM family member 5 (PGAM5) promotes mitophagy by dephosphorylating Ser13 and enhancing the FUNDC1-LC3 interaction [39]. Unc-51 like autophagy activating kinase 1 (ULK1) is induced by hypoxia or mitochondrial depolarization, and it is directed towards the mitochondria where it phosphorylates FUNDC1 at Ser17 (near the LIR motif) and stabilizes its association with LC3, This process accelerates mitophagy [40]. Under the normoxic condition, BCL2L1/Bcl-xL, a protein containing an anti-apoptotic BH3 domain, prevents the dephosphorylation of FUNDC1 at Ser13 and mitophagy by binding to PGAM5 and inhibiting the PGAM5-FUNDC1 interaction. Under hypoxia, BCL2L1 is degraded and PGAM5 is released, which promotes Ser13 dephosphorylation and thus initiates FUNDC1-mediated autophagy. Thus, the BCL2L1-PGAM5-FUNDC1 axis plays a key role in response to hypoxia-induced autophagy [41].

##### SMURF1-mediated mitophagy

SMURF1 is a homolog of the E6-AP carboxyl terminus (HECT) and a ubiquitin-like ligase that is primarily involved in the ubiquitination and breakdown of intracellular Smad proteins as well as the control of osteoblast activity [18]. SMURF1 initiates mitophagy by interacting with autophagosomes through the C2 domain [42]. Knockdown of SMURF1 significantly inhibits CCCP-induced mitophagy, without affecting the non-selective autophagy. It is discovered that the C2 domain is necessary for SMURF1-mediated mitophagy but that ubiquitin ligase activity is not. SMURF1 with a mutated C2 domain can still be recruited by damaged mitochondria, despite the fact that the production of autophagosomes and the isolation membrane’s ability to engulf mitochondria are compromised. It is suggested that SMURF1 interacts with autophagosomes through the C2 domain [42].

##### Others

**Fig. 2:**
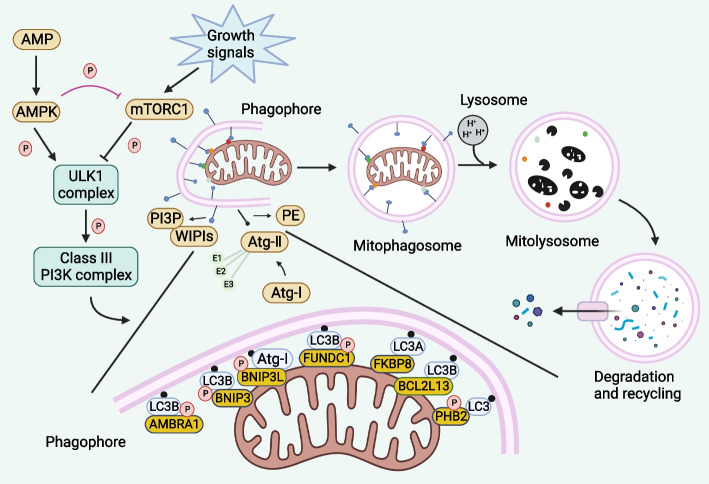
Parkin-independent pathway of mitophagy. The OMM contains a variety of proteins, including BNIP3, BNIP3L, FUNDC1, BCL2L13, FKBP8 and PHB2 as well as other autophagy receptors with LIR motifs that are not subject to ubiquitination. Direct binding of lipidized LC3 and GABARAP family members on the phagosome membrane initiates mitophagy

There are additional proteins and receptors that are in charge of mediating mitophagy in addition to the aforementioned processes for its initiation. An IMM protein called prohibitin 2 (PHB2) controls the IMM protease PARL and stops it from cleaving PGAM5. The unaltered PGAM5 can further stabilize PINK1 to recruit Parkin and other mitochondrial receptors like NDP52, thus promoting mitophagy [5]. In addition, Mitophagy is induced by BCL2L13 through a mechanism that is not dependent on Parkin [43]. BCL2L13 localizes on the OMM and directly binds to LC3 through the LIR motif to start mitophagy. A crucial modulator of cellular redox balance, NRF2 may also have an impact on mitochondrial activity. NRF2 protects mitochondrial metabolism by enhancing the stability of mitophagy via counteracting the Warburg effect (Fig. 2) [44].

### ER-phagy

ER is a tunnel system surrounded by membrane in cells, which is a crucial organelle found in cells. Sheets and tubules make up the structural components of the ER. The ER can be classified into two distinct categories: rough ER and smooth ER. The rough ER is also called the granular ER, ribosomes attached to the surface of the rough ER are the site of protein synthesis. The rough ER serves both as a transport channel for newly synthesized proteins and as a scaffold for ribosome attachment. Smooth ER is also known as non-granular ER. The cyst wall of the smooth ER has a smooth surface and no ribosome attachment. Therefore, smooth ER is not related to protein synthesis, but its function is more complex. It may be involved in the synthesis of glycogen and lipids, the synthesis of steroid hormones, and secretion [45].

The ER is a essential organelle for signal transduction and cellular metabolism. ER stress is brought on by misfolded proteins and aggregates that build up in the ER lumen under stressful or unfavorable circumstances. ER stress activates two key quality-control mechanisms: ER-associated degradation (ERAD) and the unfolded protein response (UPR) [46]. The ERAD system detects proteins that are terminally misfolded and helps them move from the ER back to the cytoplasm whereas the activation of UPR improves the ER’s ability to fold proteins. The ubiquitin-proteasome system then carries out the degradation of misfolded proteins in the cytoplasm. In order to reduce overall translation, the PERK-eIF2 signaling pathway is activated when UPR induces a variety of regulatory molecules that detect the increase of gathered and unfolded proteins in the lumen. Additionally, PERK-eIF2α signaling can activate the transcription of genes involved in the ER stress response and maintain the homeostasis and health of the ER. Excessive ER fragments are broken down by ER-phagy, which is triggered by the buildup of aggregated or misfolded proteins in the ER lumen.

**Fig. 3:**
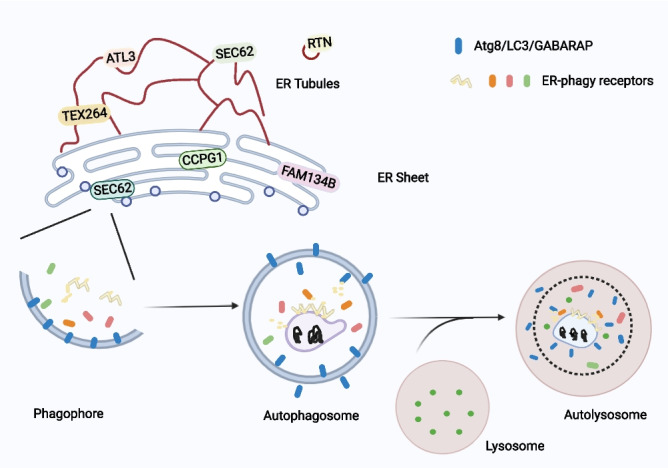
ER-phagy’s catabolic process. According to LC3/GABARAP/Atg8, ER-phagy receptors are localized in ER subdomains that need to be degraded. The endoplasmic subdomain and the autophagy mechanism are connected in this way. The isolation membrane connects to the ER assembles and expands into phagocytes. The phagosome then encapsulate the ER fragment and seal to form autophagosome. The autophagosome and lysosome then combine to produce a vacuole in yeast and plants, or an autolysosome in mammalian cells. Eventually, the components swallowed by the autophagosome are broken down by lysosome/vacuolar hydrolase

At present, a total of 11 ER-phagy receptors have been reported, including Atg39 and Atg40 in yeasts, and FAM134B, RTN3L, SEC62, CCPG1, ATL3, testis-expressed protein 264 (TEX264), calcium-binding and coiled-coil domain-containing protein 1 (CALCOCO1), CDK5 regulatory subunit-associated protein 3 (C53) and ER-phagy receptor 1 (Epr1) in mammals. These ER-phagy receptors contain the AIM/LIR/GIM motif (GABARAP interactors). They attach themselves to Atg8/LC3/GABARAP and link the autophagosomes to the ER [47]. ER-phagy receptors are dispersed throughout several areas and are active in various ER-derived compartments. FAM134B is positioned on ER sheets in mammalian cells, where it mediates ER sheet disintegration. The tubular ER is the location of RTN3L, TEX264, and ATL3, which are in charge of their degradation. Atg39 is found on the perinuclear ER (pnER) in budding yeast cells. Atg40 is primarily found in cytoplasmic ER and cortical ER. Fission yeast cells include the soluble ER-phagy receptor Epr1, whose role is comparable to that of CALCOCO1 in humans. A soluble ER-phagy receptor called C53 is present in both plant and human cells (Fig. 3).

#### ER-phagy receptors

##### FAM134B

The first discovered ER-phagy receptor is FAM134B, sometimes referred to as RETREG1 or JK1. It is only functional throughout the ER-phagy phase, and knockdown of FAM134B does not significantly influence other types of selective autophagy and macroautophagy [48]. However, loss of FAM134B promotes the expansion of ER, leading to ER stress. On the contrary, overexpression of FAM134B causes the rupture of endoplasmic reticulum membranes (MAMs) and the formation of autophagosomes [49]. Structurally, FAM134B is an intramembrane protein located on the ER sheets. The reticular homeodomain (RHD) at the N-terminus of FAM134B promotes its fixation to MAMs, thereby inducing MAMs remodeling and bending. It has been shown that calcium/calmodulin dependent protein kinase II beta (CAMK2B) can phosphorylate the RHD of FAM134B, hence facilitating the production of oligomers [50]. For ER fragmentation, FAM134B oligomerization is essential. FAM134B has an LIR motif at the C-terminus that interacts with LC3/GABARAP [49]. ATL2 is a GTPases present in ER, which may mediate the clearance of impaired ER downstream of FAM134B. Additionally, with Calnexin’s assistance, FAM134B can use ER-phagy to get rid of misfolded procollagen.

##### RTN3

RTN3 is concentrated in highly tortuous MAMs, particularly in the tubular ER. It belongs to the family of reticulons, consisting of RTN1–4 that share a highly homologous RHD but different N-terminal domains [51, 52]. Only RTN3L performs the biological activity of the ER-phagy receptor, which interacts with all 6 Atg8 family members in mammals, with GABARAP-L1 as the preferable one. The RHD at the C-terminus of GABARAP-L1 assists the anchoring of RTN3L on the ER and the bending of MAMs, and the 6 functional LIR motifs at the N-terminus form dimers that break down ER tubules into discrete fragments and transport to lysosomes [53, 54].

##### SEC62

SEC62 is located on ER sheets and tubules. SEC61, a crucial part of the translocon, is bound by the ER membrane proteins SEC62 and SEC63. Then, newly created polypeptides are transported to the rough ER [55]. Serving as an ER-phagy receptor in mammal cells, SEC62 maintains the homeostasis of ER by degrading excessive ER via triggering UPR, which is known as a process of ER remodeling or ER re-shaping. A LIR motif in the cytoplasm of C-terminus of SEC62 connects LC3 on the autophagosome membrane, thereby promoting the transition of autophagosomes toward the lysosomes. Through degrading certain ER fragments, SEC62 is able to keep the ER’s volume and dimensions [56].

##### CCPG1

CCPG1 has a role in the phagocytic destruction of ER tubules and is mostly found on the tubular ER. It consists of an N-terminus in the cytoplasm, a C-terminus and a transmembrane domain attached to the ER membrane. The C-terminal domain of CCPG1 interacts with GABARAP/LC3, and the N-terminal domain contains a LIR and two FIP200-interacting regions (FIRs), which has the ability to directly attract the FIP200-ULK complex and start autophagy [57]. In CCPG1-deficient cells, nutrient deprivation-induced ER phagocytosis impairs the RTN3-mediated tubular ER fragmentation mechanism, indicating a synergistic effect of CCPG1 and RTN3 [58].

##### ATL3

ATL3 is a member of the dynamin-like, integral membrane GTPase that is located on the tubular ER and induces the tubular ER-phagy. It includes two transmembrane helices that are tightly spaced apart and joined by a luminal polypeptide. The trans-dimerization of a GTPase domain located at the N-terminus causes the tubular ER to fuse. Two GIMs found in ATL3 target the tubular ER for lysosomal degradation by binding to GABARAP proteins [59]. A synergistic effect of ATL3 and RTN3L has been reported. In ATL3-deficient cells, RTN3L is overexpressed to compensate for the ER-phagy dysfunction, and vice versa. Additionally, ATL3 and ATL2 work together with ULK1 to encourage the complex’s recruitment to the ER and the consequent production of autophagosomes [60]. It is suggested that ATL3 exerts a dual function in ER-phagy, which not only recruits the ULK1 complex to initiate the function of phagosomes, but also induces ER fragmentation by binding to GABARAP and targets autophagosomes.

##### TEX264

TEX264 is a single-pass transmembrane ER protein, with the N-terminus and C-terminus in the lumen of the ER and the cytoplasm, respectively. It contains a LIR and an unstructured intrinsically disordered region (IDR). TEX264 exerts a vital role in ER-phagy [61].

##### Atg39

Atg39 is an ER-phagy receptor in the *S. cerevisiae*, which is localized on the pnER and served as a component of the nuclear membrane. It contains a transmembrane domain and an AIM in the cytoplasmic N-terminus. Atg39 serves a similar receptor role to that of CCPG1 in mammals [62].

##### Atg40

Atg40 is a putative yeast homologue of FAM134B that is localized on the cER and cytoER. It is responsible for the degradation of excessive cER and cytoER in the *S. cerevisiae.* Silence of Atg40 greatly blocks ER-phagy, which can be almost entirely inhibited by co-silence of Atg39 [62].

##### CALCOCO1

CALCOCO1, unlike other ER-autophagy receptors, is ER peripherally associated and is defined as a soluble ER-phagy receptor. Instead of the localization on the MAMs, CALCOCO1 targets the ER interaction with VAMP-associated proteins VAPA and VAPB on the ER membrane via a conserved fatty acid-like motif [63]. An atypical LIR motif (LVV) at the N-terminus of CALCOCO1 induces ER-phagy via binding to Atg8 family members, especially the GABARAP subfamily; whereas the C-terminus of CALCOCO1 has a UDS interface region (UIR) that can connect to UDS and help LVV bind to Atg8 family members [64].

##### C53

C53 is a cytoplasmic protein that is specifically recruited to autophagosomes in plant and mammalian cells in response to ER stress [65]. ER stress stimulates the recruitment of C53 to the ER by vesicle transportation. The C53/UFM1 specific ligase 1 (UFL1)/DDRGK domain containing 1 (DDRGK1) receptor complex is then formed to induce ER-phagy.

##### Epr1

Epr1 can mediate dithiothreitol (DTT)-induced ER-phagy. It is a soluble protein with an AIM motif and a FFAT motif in the IDR at the C-terminus. Epr1 interacts with Scs2 and Scs22, two VAPs that are present in the ER, to localize to the ER. The AIM motif attracts Atg8 to the ER during ER-phagy [66].

### Xenophagy

**Fig. 4:**
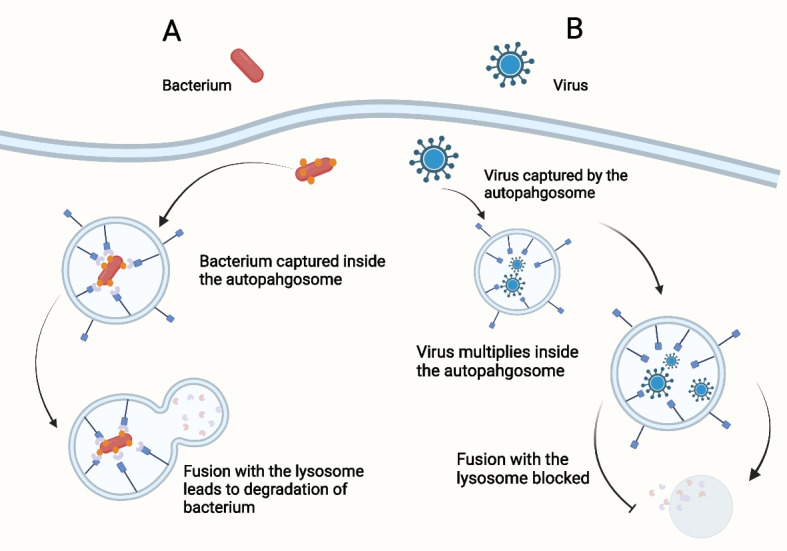
Xenophagy is a way of capturing pathogens. **A** Xenophagy captures bacteria: Ubiquitin recognizes cytoplasmic bacteria and binds to the Xenophagy adaptor protein and autophagosome membrane protein LC3. The autophagosome-containing bacteria then combines with the lysosome to breakdown. **B** Xenophagy captures the virus. The autophagosome recognizes intact viruses or virus particles, and the subsequently captures virus replicates in the autophagosome and avoids fusing with the lysosome

A crucial stage of the immune response is called xenophagy, which is a process of selective autophagy used to destroy intracellular invaders like viruses, bacteria, and fungus. By facilitating xenophagy, bacterial infection can encourage inflammation-mediated carcinogenesis [67, 68]. Normal physiological conditions result in a balance between variables that promote and inhibit inflammation. A subtle interference in the inflammatory factors or a chronic inflammation caused by the long-term infections eventually causes carcinogenesis [69]. Through recognizing, engulfing and degrading pathogens, xenophagy lowers intracellular pathogens. Thus, it is anticipated that xenophagy will serve as a cancer preventive strategy (Fig. 4).

The role of xenophagy differs from types of bacteria. Xenophagy fights against the invasion of *Streptococcus pyogenes*, serving as an innate defense system [70]. Early on in a *Salmonella typhimurium* infection, they are exposed to the cytosol within damaged vacuoles, when they are identified and targeted by heterologous autophagy. Thus it is able to prevent bacterial colonization in the cytoplasm [71]. Xenophagy exerts a protective role by inhibiting the infection of *Mycobacterium tuberculosis* [72, 73].

### Lipophagy

Lipids are naturally-occurring molecules that serve as energy supplies, signaling molecules and substrates for biological functions. Triglycerides (TG), steroids, and phospholipids are the three forms of lipids [74]. Triacylglycerol (TAG) is the correct chemical name of TG, often referred to as fat, this is the primary lipid storage or carrier. TG is usually used for food intake and lipogenesis. TG is mostly synthesized in the liver and saved in lipid droplets (LDs). The hydrolysis and metabolism of TG differ from the different parts of the body with varying fat contents. Lipid accumulation may lead to lipotoxicity, impair autophagy and lysosomal function, and thus causing diseases, metabolic syndromes or even cell death. Thus, keeping the human body’s lipid content in check is crucial to its normal operation [75, 76]. Lipophagy is a process of selective autophagy for degrading intracellular cholesterol and TG stored in LDs via the lysosomal acid lipase, which contributes to maintain the cellular lipid homeostasis by continuously recycling and re-distributing lipids [77].

In lipophagy, lysosomal lipase is expressed by Transcription factor EB (TFEB), a major transcription factor that regulates the transcription of genes involved in multiple biological pathways and is involved in important cellular functions [78]. These include autophagy, lysosomal biogenesis, lysosomal exocytosis, lipid metabolism, and mitochondrial biogenesis [79–81]. TFEB is positively correlated with the gene expression changes of autophagy genes and the relative lipidation of autophagy marker LC3. TFEB is an effective target to regulate autophagy and lysosomal activity, which can successfully combat different pathological conditions including cancer [82]. Therefore, its anti-cancer effect is worth further exploration.

**Fig. 5:**
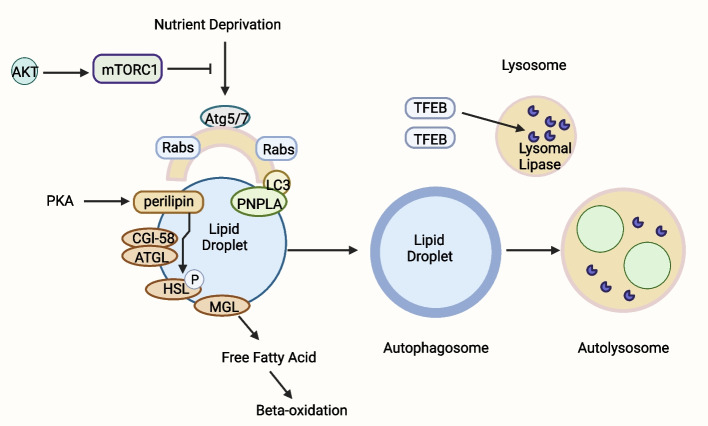
Overview of the major proteins of lipophagy. In lipolysis, TG is first hydrolyzed by adipose triglyceride lipase (ATGL) to generate diacylglycerol (DG). DG is then hydrolyzed by Hormone-sensitive lipase (HSL) to generate monoacylglycerol (MG), while HSL is phosphorylated by proteins surrounding lipid droplets. Finally, MG is hydrolyzed by Monoacylglycerol lipase (MGL) to yield glycerol and free fatty acids. Lipohagy is defined as selective autophagy degradation of LDs. In the state of nutritional starvation, lipophagy cells are formed, which are composed of Atg5, Atg7, LC3 and Rab families. The PNPLA family has specific molecular motifs associated with LDs and plays a crucial role in LDs decomposition. Autophagosome phagocytes LDs and fuses with lysosome to form autophagosome. The lysosomal lipase expressed by TFEB then hydrolyzes the neutral lipids in LDs

The receptors responsible for mediating lipid autophagy have long been a mystery, but a study by Zheng Wang et al. has filled this gap by showing that ORP8, a member of the oxysterol binding protein (OSBP) family, can act as a receptor mediating adipoautophagy [83, 84]. It is reported that the perilipin family [85–87], and Rab GTPases are closely linked with lipophagy, although the underlying mechanisms require to be further explored (Fig. 5) [88–90].

### Lysophagy

Lysosomes are major degradative organelles that degrade materials by endocytosis, phagocytosis and autophagy. Lysosomes are essential for maintaining cellular homeostasis, promoting cell growth, and performing immune defense functions [91]. The endosomal sorting complexes needed for transport (ESCRT) machinery can restore the integrity of lysosomal membranes when drugs and stimuli damage them. However, severely damaged lysosomes are cleared through a selective macroautophagic process, that is, lysophagy. Lysosomal membrane permeabilization (LMP), or complete disruption of lysosomes, is a ordinary and serious stress associated with degenerative diseases, infections, and cancers. Under normal or compromised lysosomal membrane conditions, ubiquitination of lysosomal membrane proteins attracts ubiquitination factors, including TRIM16, SKP1/CUL1/F-box protein (SCF)^FBXO27^, Leucine-rich repeat and sterile alpha motif-containing protein 1 (LRSAM1), and the ubiquitin conjugating enzyme E2Q family-like 1 (UBE2QL1). Next, the ubiquitinated protein attracts autophagy aptamers to trigger autophagy, including phospholipase A 2-activating protein (PLAA), valosin-containing protein (VCP), TAXBP-1, and SQSTM1.

**Fig. 6:**
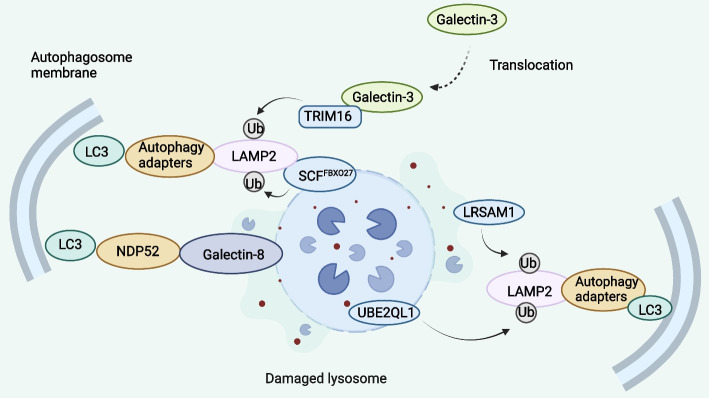
Lysophagy: Removal of damaged lysosomes by autophagy. TRIM16, UBE2QL1, SCF^FBXO27^, LRSAM1, and other lysosomal autophagy factors are brought in to ubiquitinate lysosomal membrane proteins in the event of lysosomal membrane injury or even in normal circumstances. Autophagy adapters are then recruited to induce autophagy. Galectin-3 is normally present in the cytoplasm and nucleus but can be attracted to disrupted lysosomes in the case of lysosomal damage. Phagosome formation is triggered by the assembly of autophagy initiation proteins, which is made possible by the TRIM16-Galectin-3 complex. On the other hand, Galectin-8 recruits LC3-positive phagocytes to mediate lysophagy by directly binding to the autophagy receptor NDP52, independent of ubiquitin

Galectins can also keep an eye on how the lysosomal membrane degrades and how autophagy is cleared as a result. Galactose-bound lectins are usually found in the cytoplasm and nucleus, but carbohydrate chains containing galactose are widely distributed on the cell surface and on the lumen side of the endosome, lysosome, and Golgi apparatus [92]. Damaged lysosomes undergo significant ubiquitination, which sets off the organelles’ selective autophagy. Once lysosomes are permeabilized, the binding of galectin-1 (Gal-1), galectin-3 (Gal-3), galectin-8 (Gal-8) and galectin-9 (Gal-9) to exposed β-galactosides on the inner membrane occurs and then triggers the downstream signaling pathways [93]. A synergistic effect of galectins is considered to induce lysophagy. They are able to sensitize damaged organelles, and then ubiquitin-conjugating enzyme E2Q-like protein 1 (UBE2QL1) labels lysosomal membrane proteins using K48 ubiquitin chains. After being drawn to damaged lysosomes by ubiquitin-directed AAA+ ATPase p97, p97 cofactors and YOD1 (YOD1 Deubiquitinase) remove K48-linked polyubiquitin chains. K63 ubiquitination, p62 recruitment, and binding of LC3-associated phagocytosis are accomplished by an unknown method (Fig. 6) [94].

### Pexophagy

Important metabolic enzymes for bile acid production, fatty acid β-oxidation (FAO), purine catabolism, and ether phospholipid formation are peroxisomes. They are also important redox-regulated organelles because of the dual-function of generating and scavenging reactive nitrogen species (RNS) and ROS. Therefore, maintaining proper size, number and function of peroxisomes by regulating their biogenesis and degradation is of great significance to keep homeostasis [95]. It has been established that a number of additional proteins and genes, including peroxin (PEX) and dynein-related protein 1 (Drp1), are involved in the control of peroxisome formation and division. Like other forms of selective autophagy, pexophagy necessitates certain adapters and receptors. An adapter translocates to the peroxisome membrane and links peroxidase to the autophagosome, thus inducing pexophagy. So far, 5 receptors/adaptors in eukaryotes have been identified to induce pexophagy, including Atg30 in *Pichia pastoris (P.pastoris)*, Atg36, NBR1 and p62 in *S.cerevisiae*, and acyl-CoA-binding domain containing protein 5 (ACBD5) in mammals [92].

**Fig. 7:**
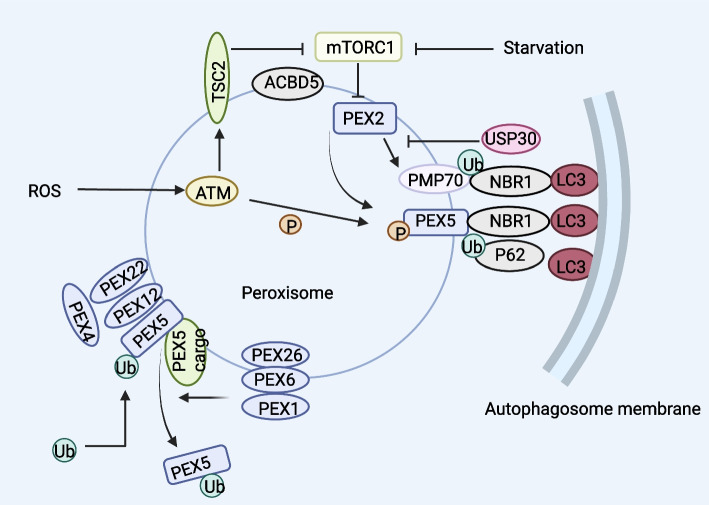
Overview of the major proteins of Pexophagy. Under normal circumstances, the mTORC1-mediated proteasome pathway can maintain low PEX2 expression levels. Under starvation conditions, an increase in PEX2 causes PEX5 and PMP70 to get ubiquitinated, which ultimately triggers Pexophagy in an NBR1-dependent way. USP30 offsets PEX2 by deubiquitinating its substrate to prevent Pexophagy. The initial response mechanism of peroxisome ROS is ATM serine/threonine kinase. TSC2 is induced by activated ATM kinase, and mTORC1 is inhibited by activated TSC2. Additionally, ATM phosphorylates PEX5 at Ser141, which causes PEX5 to be ubiquitinated at Lys209. After that, ubiquitinated PEX5 attaches itself to p62/NBR1 to trigger reactive oxygen species autophagy. The proteophage target of ubiquitin-dependent peroxisome is monobititinated PEX5 on Cys11. PEX1 and PEX6, which are affixed to the peroxisome by PEX26, will ubiquitinate PEX5 and remove it from the membrane after transit. Thus far, ACBD5 is the sole protein that is specific to phagocytic cells. Recruitment of Pexophagy-specific receptors or adapters may be facilitated by ACBD5

*P. pastoris* is a widely used model in the research of pexophagy. Atg30 is localized on the peroxisome membrane, which is transiently translocated to pre-autophagosomal structures (PAS) during the process of pexophagy. By assembling the pexophagic receptor-protein complex (RPC), it regulates pexophagy. Pexophagy is induced by overexpression of Atg30, and Atg30 phosphorylation requires peroxisomal biogenesis factor 3 (PEX3) [96]. Positive regulation of RPC assembly is provided by Atg37 and ACBD5 [97]. Atg30 selectively destroys peroxisomes through interactions between Atg8 and Atg11 and RPCs, this process is dependent on PEX3 and Atg37. In addition, the serine/threonine protein kinase HRR25 phosphorylates Atg30. Atg37 and PEX3 can regulate HRR25 positively and negatively, respectively. Because Atg37 also serves as an acyl-CoA binding protein (ACBP), acyl-CoA influences the interaction between Atg30 and Atg37, which in turn influences how Atg11 is recruited to RPC [98]. A ubiquitin ligase called PEX2 is involved in the mammalian process of pexophagy. It induces peroxisome ubiquitination and pexophagy in a way that is NBR1 dependent. Normally, mTORC1 maintains a low level of PEX2 via the ubiquitin-proteasome pathway [99], which also ensures that the peroxisomal membrane is recycled for ubiquitinated PEX5. Amino acid starvation induction upregulates PEX2, and subsequently, ubiquitinated PEX5 and 70-kDa peroxisomal membrane protein (PMP70) are degraded via pexophagy via the recruited NBR1 (Fig. 7) [100].

### Other types of autophagy

So far, 25 kinds of selective autophagy have been identified [101]. Besides the abovementioned selective autophagy, the role of novel types like nucleophagy and ferritinophagy in cancers has been gradually concerned. Nucleophagy is responsible for maintaining the nuclear integrity and genome stability during the early stages of carcinogenesis by eliminating problematic genetic materials. However, in the advanced stage, nucleophagy is favorable to cancer cell survival and metastasis [102]. It is reported that a cytosolic iron chaperone poly (rC)-binding protein 1 (PCBP1) negatively mediates ferritinophagy-induced ferroptosis by impairing the stability of BECN1 mRNA. Therefore, silencing PCBP1 is a potential therapeutic strategy for killing ferriphilic refractory cancer cells via enhancing the susceptibility to ferroptosis [103]. Moreover, studies have shown that Ubiquitin-specific peptidase 8 (USP8) plays an important role in regulating ferritinophagy and ferroptotic responses in cancer cells, revealing that USP8 could be a viable target for cancer therapies using ferroptosis [104]. Even if there are more and more forms of selective autophagy, the mechanism behind them is still unclear, posing a huge challenge on clinical research.

## Regulatory effects of selective autophagy in cancers

Carcinogenesis can be inhibited or promoted by autophagy, making it a double-edged sword. It prevents cancer cell proliferation and stabilizes the genome by degrading damaged organelles in cancer cells during the process of carcinogenesis and malignant transformation. However, in the malignant microenvironment, autophagy is essential for cancer development and progression to provide energy and nutrients [105].

### Mitophagy in cancers

#### Carcinogenic effect of mitophagy

##### Mitophagy provides sufficient nutrients, energies and oxygen to cancer cells

**Fig. 8:**
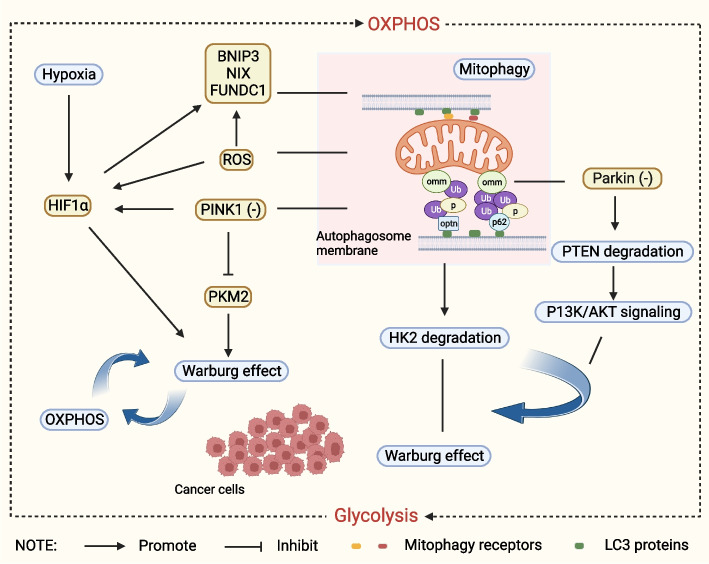
Mitophagy provides sufficient nutrition, energy and oxygen for cancer cells. The Warburg effect is the process by which glycolysis-a process that converts glucose into lactic acid-is promote by mitophagy. Mitochondrial OXPHOS and glycolysis act in concert to maintain the balance of energy metabolism in cancer cells. Parkin loss stimulates PTEN degradation, which in turn sets off the typical carcinogenic pathway known as PI3K/AKT signaling. This route expedites cancer cells’ aerobic glycolysis. Similarly, PINK1 absence can cause the Warburg effect by lowering Pyruvate kinase M2 (PKM2) activity and stabilizing HIF1a, which keeps cancer cells proliferating quickly. PKM2 is one of the key enzymes in glycolysis, and reducing PKM2 activity can promote the rapid proliferation of cancer cells by stimulating the pentose phosphate pathway. Moreover, hexokinase 2 (HK2) is selectively degraded by p62/SQSTM1-dependent mitophagy to control glycolysis levels. HIF1a transcription is up-regulated, initiating glycolytic metabolism, and its target genes are expressed more, which controls mitophagy. Hypoxia stimulation and the increase and elevation of mitochondrial ROS also cause these effects

A defining feature of the tumor microenvironment, hypoxia can accelerate the spread of cancer. In the initial stage of carcinogenesis, a microenvironment formed by rapidly proliferating cancer cells and local hypoxia and nutrient deficiency causes mitochondrial dysfunction and thus induces mitophagy. Cancer cells need a demand of more nutrients, and mitophagy provides amino acids for cell growth via recycling from lysosomes. Mitophagy not only provides nutrients for ATP production and biogenesis, but also satisfies metabolic needs of cancer cells by degrading carbohydrates, proteins, lipids and nucleotides [106]. In order to maintain uncontrolled growth rates, cancer cells employ unconventional mechanisms to obtain energy from the outside world. Due to mitochondrial dysfunction, mitochondrial oxidative phosphorylation (OXPHOS) is inhibited in cancer cells. This reprogramming of energy metabolism is known as the Warburg effect [107]. Mitosis promotes the glycolytic pathway and reduces the use of OXPHOS mechanism, which facilitates OXPHOS to meet the rapidly increasing energy demand. Mitochondrial OXPHOS and glycolysis act synergistically to maintain the balance of energy metabolism in cancer cells [108]. Additionally, mitophagy can inhibit the production of ROS and an ineffective consumption of valuable nutrients like oxygen, which promotes the fast growth of cancer cells (Fig. 8) [109].

##### Mitophagy maintains the stem cell properties of cancer stem cells (CSCs)

**Fig. 9:**
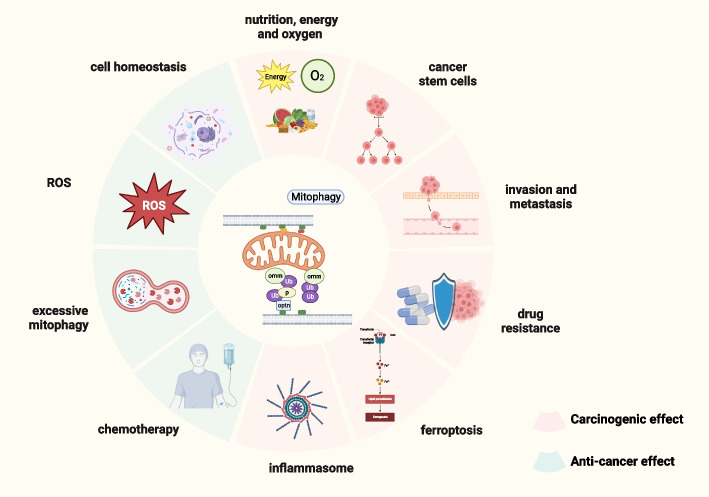
The dual role of Mitophagy in cancer. On the one hand, mitophagy can promote cancer by providing adequate nutrition, energy and oxygen to cancer cells, maintaining the stem cell characteristics of tumor stem cells, promoting the invasion and metastasis of cancer cells, mediating drug resistance of cancer cells, inhibiting iron death, and activating inflammasome. On the other hand, mitophagy at the basal level can degrade dysfunctional mitochondria to maintain cell homeostasis, limit the production of ROS and thus inhibit cancer. And excessive mitophagy and mitophagy after chemotherapy can also promote cancer death

Mitophagy is crucial for maintaining the stem cell features of CSCs [110]. Through switching from OXPHOS to glycolysis, essential properties of CSCs are maintained, including stem cell self-renewal and cancer growth. To satisfy the energy requirements, a metabolic remodeling of CSCs is achieved via mitophagy, which stimulates metabolic reprogramming, the acquirement of the glycolytic or OXPHOS phenotype, and the progression of cancer (Fig. 9) [111].

##### Mitophagy promotes cancer cell invasion and metastasis

In order to prevent metastasis, when anchorage-dependent cells split out from the extracellular matrix around them, apoptosis takes place. This process is known as anoikis [112]. The capacity to resist anoikis has been evolved by cancer cells with malignant potential, thus being survived after detaching the primary lesion and metastasizing through the lymphatic or circulatory system. There is mounting evidence that mitophagy protects cancer cells against anoikis. Additionally, mitophagy reduces the possibility of apoptosis by maintaining mitochondrial functions (such as ATP synthesis, antioxidant defense, and prevention of DNA damage) and energy balance (Fig. 9) [113].

##### Mitophagy mediates drug resistance in cancer cells

Drug resistance significantly limits chemotherapy’s ability to treat patients. Cancer cells can induce resistance through mitophagy. It is reported that mitophagy activates autophagy in the neuroblastoma cell line SH-N-AS, thus enhancing the resistance to the anti-cancer agent UNBS1450 [114]. By inhibiting mitophagy, the isoquinoline alkaloid lentinine lowers breast cancer cells’ susceptibility to common chemotherapy medicines [115]. It is suggested that mitophagy may increase cancer cells’ resistance to chemotherapy, and inhibiting mitophagy is a promising approach to improve the anti-cancer effect. A comprehensive understanding of tumor-related signaling pathways and the physiological functions of autophagy is expected to open up new possibilities for the treatment of tumor drug resistance and the improvement of clinical outcomes [116] (Fig. 9).

##### Mitophagy promotes cancer cell survival by inhibiting ferroptosis

Ferroptosis is a type of programmed cell death characterized by the dependence on irons and accumulation of lipid peroxides [117]. Nuclear factor erythroid 2-related factor 2 (Nrf2) can directly or indirectly regulate many genes related to ferroptosis, which uses both Parkin/PINK1-independent and p62-dependent mitophagy to control the dynamics of the mitochondria. Moreover, the activation of Nrf2 strengthens cancer cells’ resistance to ferroptosis [118]. It is suggested that mitophagy helps cancer cells survive by preventing ferroptosis via activating Nrf2. Nevertheless, the exact interaction between mitophagy and ferroptosis has not been fully elucidated (Fig. 9).

##### Mitophagy promotes carcinogenesis by activating inflammasomes

It is validated that mitophagy exerts its biological role in cancers through activating the inflammasomes [119]. During the process of mitophagy, the deficiency of PINK1 and PARK2 activates the AIM2 inflammasome and thus accelerates the aggravation of pancreatic cancer [120]. In mitophagy receptor FUNDC1-deficient hepatocytes, the accumulation of dysfunctional mitochondria causes the activation of inflammasomes, overproduction of IL-1β and hyperproliferation of hepatocytes [121]. Therefore, carcinogenesis may be influenced by inflammasome activation and mitochondrial homeostasis abnormalities (Fig. 9).

#### Anti-cancer effect of mitophagy

##### Basal level mitophagy acts as a tumor-suppressing mechanism by reducing damaged cell components and proteins and preserving cell homeostasis

Mitophagy is also thought to be an anti-tumor mechanism [122]. Mitophagy is suppressed and damaged mitochondria aggregate as a result of some genes dysfunction. This promotes the development of tumors. Growing data from numerous studies lends credence to the idea that adaptor proteins, or certain mitotic receptors, function as tumor suppressors in cancer. For example, the Parkin/PARK2 gene is situated at the vulnerable location of chromosome 6 q25.2-q27 and carries mutations [25] in lung cancer [123], breast cancer [124], glioma (Fig. 9) [124].

##### Mitophagy can degrade dysfunctional mitochondria, limit ROS production, and inhibit cancer

In mice, Parkin or PINK1 deletion promotes KRAS-driven pancreatic carcinogenesis [120] and results in the development of hepatocellular carcinoma [125]. In humans, Parkin deletion has been found in tumors including colorectal cancer [126], glioblastoma [27], melanoma [127], lung cancer [128], and breast cancer [129]. Increased pro-inflammatory signaling, genomic instability [128], and increased cancer cell proliferation and resisitance to apoptosis [125] are all caused by parkin deletion. Due to the buildup of mitochondrial malfunction brought on by Parkin loss, ROS generation, glycolysis, and mitochondrial OXPHOS are all increased. This may contribute to the Warburg effect and hence encourage the growth of malignancies (Fig. 9) [130].

##### Excessive mitophagy leads to cancer cell death

Under the normal circumstance, mitophagy is a defensive mechanism to protect cells through degrading damaged mitochondria. Nevertheless, an excessive mitophagy results in the abnormal mitochondrial cycle and energy metabolism disturbance, finally leading to cell death. Hypoxia-inducible factor 1-alpha (HIF1A), which is activated by the growth of cancer cells, causes hypoxia and upregulates BNIP3. Pro-apoptotic molecule BNIP3 promote mitophagy, which inhibits the fusing of damaged mitochondria [131]. It is reported that ceramide-induced the upregulation of BNIP3 in glioma cells, which further activates mitophagy and causes cancer cell death (Fig. 9) [132].

##### Mitophagy promotes cancer cell death after chemotherapy

An excessive mitophagy causes the type II programmed cell death. Existing evidences have shown that one of the main mechanisms behind cancer cell death is autophagy-dependent cell death. After chemotherapy, mitophagy contributes to inhibit the progression of cancers by accelerating cancer cell death [133]. Emeric Limagne et al. found that Adding a MEK inhibitors to pemetrexed-cisplatin can promotes mitophagy, thereby restoring the efficacy of chemoimmunotherapy in cancer treatment [133]. Mohammed Dany et al. found that LCL-461 reduced resistance to the anti-acute myeloid leukemia drug crenolanib by inducing mitophagy [134]. It is concluded that triggering mitophagy in cancer cells may be useful anti-cancer treatment (Fig. 9) [16].

### ER-phagy

In carcinogenesis, ER-phagy plays a complicated role. Because ER-phagy lessens excessive stress in the ER, cancer cells are better equipped to proliferate and survive. On the contrary, ER-phagy is also an anti-cancer mechanism to induce cancer cell death. The exact role of ER-phagy in cancers depends on the cancer types, stage of progression and the microenvironment [135].

#### Carcinogenic effect of ER-phagy

There are two roles for FAM134B in cancer. Firstly, Cancer growth may be aided by FAM134B-mediated ER-phagy. FAM134B acts as a tumor promoter in esophageal squamous cell carcinoma and hepatocellular carcinoma [136, 137]. Another study on colorectal cancer found that ER-phagy mediated by FAM134B can reduce UPR induced by treatment drug brigatinib, thus promoting the survival of cancer cells, while knockdown of FAM134B can increase the sensitivity of colorectal cancer to brigatinib [6].

High levels of SEC62 expression have been associated with increased resistance to UPR and other endoplasmic reticulum stress in non-small cell lung cancer and thyroid cancer cells, which facilitates cancer cell invasion and migration [138]. Thyroid, prostate, and NSCLC cancer cells were more susceptible to ER stress brought on by thapsigargin after SEC62 expression was inhibited. In HeLa cells, suppressing SEC62 likewise stops the cells from migrating. These combined observations imply that SEC62-mediated ER-phagy may aid cancer cells in better coping with endoplasmic reticulum stress and aid in their survival and migration [139].

According to earlier studies, the N-terminal R12H of CALCOCO1 is associated with colorectal cancer metastasis and breast cancer development [123, 124]. The N-terminal R12H mutation of CALCOCO1 reduces the interaction between CALCOC1 and LC3C. Consequently, CALCOCO1 mutation-induced ER-phagy deficiency may be associated with the development of breast cancer [135].

By controlling the key tumor suppressors p53 and CDK1 cyclin B1 [140], the development of cancer cells is inhibited by C53. On the other hand, it has also been demonstrated that significant levels of C53 expression are present in hepatocellular carcinoma cell lines, indicating that this protein may be involved in promoting tumor invasion and metastasis [141]. When protein translation is obstructed, ER stress can be responded by ER-phagy mediated by C53. Thus, we hypothesized that C53 helps cancer cells survive ER stress by upregulating ER autophagy [142].

#### Anti-cancer effect of ER-phagy

Autophagic cell death can be caused by excessive ER-phagy in cancer cells, which is mediated by FAM134B. Z36 is a tiny molecule that has been shown to cause cancer cell death by promoting excessive ER-phagy and inducing the expression of FAM134B in HeLa cells [143]. It has been shown that FAM134B can prevent the development of colorectal and breast cancer [144, 145]. The suppressive effect of FAM134B on cancer needs to be further studied and tested in clinical practice.

### Xenophagy

It has long been known that infections play a major part in the growth of sporadic malignancies caused by genomic instability or DNA damage. These conditions are associated with the generation of toxic metabolites and chronic inflammation mediated by pathogens. Through interacting and activating oncoproteins of the host, bacterial effectors contribute to cell cycle dysregulation and thus carcinogenesis [69]. Cell death mediated by xenophagy is favorable to tumor regression. It can also serve as a protector to slow down tumor growth by preventing bacterial infection [146, 147]. Since bacteria-associated xenophagy is able to influence the microbiota and then triggers carcinogenesis via stimulating inflammation, antimicrobial agents may have the potential to prevent cancers by regulating xenophagy [148].

#### Carcinogenic effect of xenophagy

The main factor contributing to stomach cancer carcinogenesis is an infection with *Helicobacter pylori (H. pylori)*. Cytotoxin A (VacA) and cytotoxin-associated gene A (CagA) are the two main bacterial proteins that *H. pylori* uses to regulate gastric epithelial cells. An acute exposure to VacA prevents *H. pylori* infection by inducing xenophagy, while a long-term exposure strongly disrupts xenophagy, promotes infection and eventually causes carcinogenesis by upregulating SQSTM1, and increasing the accumulation of ROS and toxins [149]. There is a close correlation between the carcinogenesis of gastric cancer and the continuous expression of CagA. Infected cells generally undergo xenophagy triggered by ROS to breakdown CagA [68]. *peptidoglycan deacetylase (PgdA)* is essential for controlling the inflammatory response to *H. pylori* infection by reducing NOD1-dependent activation of NF-κB and inhibiting xenophagy, which eventually induces gastric cancer [150]. Xenophagy is a key mechanism in recognizing *H. pylori* and inducing *H. pylori*-associated gastric cancer. The xenophagy in cells is significantly suppressed by the extremely pathogenic *H. pylori* strain GC026. The rs2241880 mutation in the autophagy-related 16-like 1 (Atg16L1) gene is associated with higher incidence of gastric cancer and *H. pylori* infection, which implies that defective xenophagy may be involved in the carcinogenesis of gastric cancer [146].

#### Anti-cancer effect of xenophagy

In melanoma cells, *murine typhus* stimulates xenophagy via suppressing the Akt-mTOR-RPS6KB/p70S6K signaling pathway [151]. When *Salmonella* accumulatively lives in malignant lesions, strong xenophagy is induced by cancer cells in order to eradicate the bacteria through LC3 processing. In hepatocellular carcinoma cells, tumor-targeting *Salmonella typhimurium* A1-R or the strain VNP20009 of the bacterium causes xenophagy, which wards off infection by cancer cells [147]. Knockdown of Atg5 or BECN1 in cancer cells infected with bacteria significantly increases bacterial proliferation and slows down cancer cell growth [147]. Therefore, a combination therapy of *Salmonella*-targeted xenophagy blockade and anti-infection treatment is a promising anti-cancer strategy.

### Lipophagy

#### Carcinogenic effect of lipophagy

Lipophagy is an alternative of lipid droplet degradation, which is a key factor for carcinogenesis and metastasis by mediating lipid turnover. Cancer cells typically encourage the synthesis and uptake of fatty acids, which causes the production of lipid droplets [152]. In times of stress or nutrient deprivation, lipophagy supplies lipid metabolites for the synthesis of macromolecules, which may contribute to cancer cell survival [153]. For example, lysosomal acid lipase (LAL) inhibition helps prevent prostate cancer by preventing the creation of free fatty acids and reactive oxygen species (ROS) that are produced as a result of lipase activity [154]. Suppressing lipophagy is linked with increased cancer aggressiveness [155, 156] and chemotherapy resistance [157]. LAL deficiency results in hematopoietic abnormalities. Then, immunological evasion and cancer cell metastasis are made possible by massive immature myeloid-derived suppressor cells (MSDCs), which act as a mediator in the immune surveillance suppressing [158, 159].

#### Anti-cancer effect of lipophagy

Enhancement of lipid metabolism can alleviate the metastasis of lung and liver cancer [136]. According to a study, lipophagy mediates ER stress by accumulating free fatty acids, which makes cancer cells more susceptible to death [160]. As a type of lipid metabolism, lipophagy is considered as a promising anti-cancer treatment.

### Lysophagy

Lysosomes contain hydrolytic enzymes like cathepsins that degrade proteins during autophagy. Macrocytosis that relies on the degradation of extracellular materials via lysosomes is stimulated in nutrient-deficient cancers [161, 162]. Lysosomes are involved in drug resistance by blocking anti-cancer agents to their target molecules [94]. By releasing hydrolases such as cathepsins from the lysosomal lumen into the cytosol, LMP causes necrosis or death in cells. It is an interesting process that may prevent carcinogenic effect of apoptosis as the main cell death mechanism. LMP induction appears to be a successful method of killing cancer cells, given the critical roles that functional lysosomes play in drug resistance and cancer cell survival.

### Pexophagy

Peroxisomes play a vital role in the metabolism of cancer cells by oxidizing several kinds of chemicals, including fatty and amino acids. Although the specific role of peroxisomes in cancers has not been highlighted, their increased activities may promote the malignant growth via lipid oxidation [163]. Acetyl-CoA oxidase 1 (ACOX1) and other peroxidase metabolism-related genes can have their expression levels controlled by peroxisome proliferator-activated receptors (PPARs) [164]. Hepatocellular carcinoma is associated with an upregulation of ACOX1, which promotes the liver’s oxidation of fatty acids and the production of H2O2, thereafter favoring the malignant development [165]. A poorer prognosis for HER2-positive breast cancer is associated with high levels of ACOX1 [166]. Alpha-methylacyl-CoA racemase (AMACR) participate in the α-oxidation of molecules and the preparation for β-oxidation. The overexpression of AMACR is associated with low survival of prostate cancer [167], colon cancer [168], gastric cancer [169], breast cancer [170], renal and hepatocellular carcinoma [171], and myxofibrosarcoma [152].

## Small molecule compounds targeting selective autophagy for cancer therapy

Autophagy can act as a promoter or inhibitor of cancer, which makes it a promising and challenging therapeutic target [172]. At present, only chloroquine (CQ) and its derived hydroxychloroquine (HCQ) are FDA-approved drugs to inhibit autophagy. However, its low potency may limit its anti-tumor efficacy. Based on this, a series of chloroquine analogues have been synthesized, which retain the incorporation of 4-aminoquinoline subunits into different substituents triazoles into the target structure. The most potent of these compounds, EAD1, proved to be a viable lead compound for evaluating the anti-tumor activity of autophagy inhibitors in vivo [173]. More and more small molecule compounds that target selective autophagy pathways have been made available recently as a result of rational drug design and screening. These compounds can be employed as a basis for drug development or as tools for study. In this review, we summarize the information of some small molecule compounds targeting selective autophagy as well as different active autophagy drugs that have been developed in clinical trials. However, no clear drugs targeting selective autophagy have entered clinical trials and deserve our further exploration. It is hoped to provide new clues for the regulation of autophagy as an adjuvant treatment strategy to conquer cancer (Tables 1 and 2).

**Table 1:** Small molecule compounds targeting selective autophagy for cancer therapy

Selective autophagy type	Name in the literature	Chemical structure	Cancer	Target	Biological activity	Ref
mitophagy	WJ460		breast cancer, pancreatic ductal adenocarcinoma (PDAC)	Myoferlin	MiaPaCa-2 (IC_50_ = 20.92 ± 1.02 nM)), BxPC-3 (IC_50_ = 48.44 nM), Panc-1 (IC_50_ = 23.08 ± 1.08 nM), PaTu 8988 T (IC_50_ = 27.48 nM)	[4]
mitophagy	flavaglines compound 3 (FL3)		^a^ NA	PHB2	HeLa, H1299, HCT116 (IC = 50 nM)	[5]
mitophagy	fluquinconazole		lung cancer, cervical cancer	PHB1, PHB2	A549 (IC = 5 μM, 10 μM), HeLa (IC = 5 μM, 10 μM)	[174]
mitophagy	Nitazoxanide (NTZ)		bladder cancer	PINK1	MGHU3 (IC_50_ = 54.87 ± 2.59 μM), 5637 (IC_50_ = 72.28 ± 3.47 μM), T24 (IC_50_ = 57.62 ± 2.08 μM), UMUC-3 (IC_50_ = 76.74 ± 3.36 μM)	[175]
mitophagy	oroxylinA (OA)		hepatocellular carcinoma	Parkin/PINK1	HepG2 (IC = 12 μM)	[150, 151]
ER-phagy	loperamide (LOP)		glioblastoma multiforme	RETREG1/FAM134B	MZ-54 (IC = 17.5 μM)	[176]
ER-phagy	brigatinib		non-small cell lung cancer	ORP8/USP5	DLD-1, HCT116, HT29, RKO, SW620, NCM460 (IC = 2 μM)	[6, 177]
ER-phagy	C150		pancreatic cancer	EMT- TF	PANC-1 (IC = 1, 2 μM)	[178]
ER-phagy	ABTL0812		neuroblastoma	AKT/mTOR	SH-SY5Y(IC_50_ = 30–60 μM)	[179–182]
ER-phagy	Z36		cervical cancer	FAM134B/LC3Atg9	HeLa (IC = 13 μM)	[183]
Xenophagy	Resveratrol		squamous cell carcinoma, oral cancer	P53,AMPK	HeLa, HCT116 (IC = 130 μM), CAR (IC_50_ = 51.62 ± 3.36 μM)	[184–186]
Xenophagy	Tigecycline		gastric cancer, Melanoma	AMPK/mTOR/p70S6K,LC3A/B	MKN-45, GAM-016 (IC = 5 μM, 10 μM), COLO 829 (EC_50_ = 19.2 μM), A375 (EC_50_ = 39.1 μM)	[184, 187]
Xenophagy	Salinomycin		osteosarcoma	^a^ NA	U2OS (IC_50_ = 5 μM)	[188]
Lipophagy	Tripterine		clear cell renal cell carcinoma (ccRCC)	LXRα/ABCA1	786-O (IC = 1 μM), 787-SN12C (IC = 1 μM)	[189]
Lipophagy	PFK158		ovarian and cervical cancer	PFKFB3/p62/SQSTM1	OV2008 (IC_50_ = 10 μM), C13 (IC_50_ = 10 μM), HeyA8 (IC_50_ = 10 μM), HeyA8MDR (IC_50_ = 10 μM)	[190]
Lysophagy	loperamide		glioma	SMPD1/ASM	MZ-54 (IC = 12.5,15 μM)	[191]
Lysophagy	Pimozide		glioma	SMPD1/ASM	MZ-54 (IC = 12.5,15 μM)	[191]
Lysophagy	GNS561		hepatocellular carcinoma	PPT1	HepG2 (IC_50_ = 0.47 ± 0.15 μM), Huh7 (IC_50_ = 0.88 ± 0.31 μM),	[192]

**Table 2:** Small molecule drugs targeting autophagy have entered clinical trials

Drug name	Chemical structure	Phase	Cancer	Clinical trials identifier	Ref
CQ		I	glioblastoma	NCT02378532	[193]
HCQ		II	Non-Small Cell Lung Cancer	NCT04735068	[194]
ABTL0812		I	solid tumours	NCT02201823	[195]
Sirolimus		I	Lymphangioleiomyomatosis	NCT01687179	[196]
temsirolimus		I	melanoma	NCT00281957	[197]
Pantoprazole		II	Metastatic Castration-Resistant Prostate Cancer	NCT01748500	[198]
2-OHOA		I	glioma	NCT01792310	[199]
Neratinib		I	pancreatic cancer	NCT02349867	[200]

^a^ *NA* Not available

### Targeting mitophagy

#### WJ460

A membrane-anchored protein called myoferlin is overexpressed in several cancer types. It is a developing target for mitophagy-based anti-cancer therapy [148, 149]. WJ460 is a pharmacologically active compound targeting myoferlin. It triggers mitophagy and induces accumulation of ROS by targeting myoferlin in pancreatic ductal adenocarcinoma (PDAC), which eventually causes lipid peroxidation and ferroptosis. In addition, the synergistic effect of WJ460 with ferroptosis inducers erastin or RSL3 can enhance the ferroptosis outcome of cancer cells [4]. Hence, a combination treatment of agents targeting myoferlin and low-dose ferroptosis activators can effectively kill PDAC cells.

#### FL3

A novel inner mitochondrial membrane mitophagy receptor has been recognized as PHB2, a highly conserved membrane scaffold protein [5]. The PHB protein ligand FL3, at nanomolar concentrations, has been shown by Yan et al. to strongly block PHB2-mediated mitophagy, as well as to stop cancer cell proliferation and energy production. In HeLa cells expressing GFP-Parkin, the degradation of mitochondria and the mitochondrial recruitment of Parkin and the accumulation of PINK1 in polarized mitochondria were significantly inhibited by FL3 treatment for 24 h followed by the induction of mitophagy. It has been proposed that FL3 targets PHB2 specifically in order to influence Parkin/PINK1-mediated mitophagy. Furthermore, the proliferation of several cancer cell lines, such as the cervical cancer cell line HeLa, the p53-null NSCLC cell line H1299, and the wild-type p53 CRC cell line HCT116, is markedly inhibited by a low dose of FL3 (50 nM). FL3 exerts a potent anti-cancer effect in vivo without causing major adverse events, and therefore, targeting PHB2 is a promising anti-cancer therapy [5].

#### Fluorizoline

PHB1 and PHB2 are significant mitophagy receptors that facilitate the autophagic breakdown of mitochondria. Fluorizoline inhibits Parkin-dependent as well as Parkin-independent mitophagy by directly targeting PHB1 and PHB2 in A549 and HeLa cells that are stable Parkin expression cultures. This suggests that fluorizoline is a potential anti-cancer drug through mitophagy regulation [177].

#### Nitazoxanide (NTZ)

NTZ damages mitochondria and triggers mitophagy in a three-dimensional in vitro cell culture model by using autophagy receptors and phosphoubiquitin (pS65-Ub) produced by PINK1. NTZ impairs mitophagic flux in the late stage by inhibiting lysosomal degradation activity, which can be further aggravated by the combination treatment with the autophagy inhibitor CQ. Studies have shown that NTZ significantly inhibits orthotopic bladder tumors without causing an obvious systemic toxicity, suggesting that NTZ exerts the anti-cancer activity at different stages through ROS-mediated mitophagy. NTZ is believed as a potential agent against bladder tumors [175].

#### Oroxylin a (OA)

A new CDK9 inhibitor called OA was isolated from Scutellaria baicalensis [201]. Yao et al. found that OA has a strong therapeutic potential in hepatocellular carcinoma (HCC), which inhibits Parkin/PINK1-mediated mitophagy to overcome drug resistance. By suppressing CDK9, downregulating PINK1, and restricting Parkin’s recruitment to mitochondria, OA stops mitophagy from starting. Hence, OA is a mitophagy inhibitor that exerts the anti-cancer role in HCC [202].

### Targeting ER-phagy

#### Loperamide (LOP)

Svenja Zielke et al. demonstrated that LOP upregulates stress signaling transcription factor ATF4 to induce ER stress, thereby inducing autophagy, ER-phagy and autophagic cell death (ACD). Experiments showed that LOP-induced ER-phagy is mediated by RETREG1 and TEX264 ER-phagy receptors. The degradation of rough endoplasmic reticulum fragments was mainly targeted. In glioblastoma cells, ATF4 and ER stress are primary regulators for LOP-induced ER-phagy, and ER-phagy and autophagic cell death (ACD) induced by RETREG1 and TEX264. As the basis for LOP-induced ER-phagy and its possible interaction with cancer cell death, future studies are needed to identify other ER-phagy receptors, cofactors, and regulatory mechanisms [176].

#### Brigatinib

An anaplastic lymphoma kinase (ALK) inhibitor called brentinib is used to treat ALK-positive non-small cell lung cancer [177]. In CRC, Zhang et al. found that brigatinib had an ALK-independent anti-cancer mechanism. After treatment with Brigatinib on CRC cell lines HT29, RKO, SW620 and the human colonic mucosal epithelial cell line NCM460, brigatinib was found to trigger CRC by inducing ER stress mediated by oxysterol-binding protein-related protein 8 (ORP8) /ubiquitin-specific peptidase 5 (USP5) apoptosis, and brigatinib was also found to induce FAM134B-mediated ER-phagy. The anti-cancer activity of brigatinib on colorectal cancer is boosted when combined with autophagy inhibitors such as CQ, 3-methyladenine (3-MA), or bafilomycin A1. It is suggested that brigatinib is a promising anti-cancer drug for the treatment of CRC [6].

#### C150

In pancreatic cancer, Tao Wang et al. identified a potential drug called C150 that inhibits the process of epithelial-to-mesenchymal transition (EMT) and also provided information about the way in which this molecule functions. In human pancreatic cancer cell lines PANC-1 and MIA-PaCa-2 cells, as well as murine pancreatic cancer line Pan02, C150 causes ER stress and subsequently increase the assembly and activity of proteasomes and the degradation of transcription factors involved in the epithelial mesenchymal transition (EMT-TF). Pancreatic cancer cells exhibit inhibition of protein synthesis in addition to response to ER stress and enhanced ER-phagy. Moreover, C150-induced ER-phagy causes cell cycle arrest in G2/M phase, which prevents pancreatic cancer cells from proliferation and induce senescence. PANC-1 cells’ susceptibility to gemcitabine is further increased by C150-induced cell senescence. Therefore, there is a lot of promise for treating pancreatic cancer with the combination of C150 and gemcitabine [178].

#### ABTL0812

The sodium salt formulation known as ABTL0812 is derived from 2-hydroxy-linoleic acid, a significant polyunsaturated omega-6 fatty acid with 18 carbons. Through the inhibition of the AKT/mTOR pathway, sustained induction of ER stress, and activation of the UPR, ABTL0812 induces cytotoxic autophagy [179]. The stability or expression of MYCN, which is a crucial non-druggable genetic driver in neuroblastoma, can be regulated by the AKT/mTOR pathway and ER-phagy [180, 181]. In the phase I/Ib trial (NCT02201823), safety and tolerability of ABTL0812 have been validated [182]. It induces neuroblastoma cell death by activating ER stress, UPR, apoptosis and autophagy. Meanwhile, 13-cis retinoic acid and irinotecan are two examples of differentiation and chemotherapeutic drugs whose anti-cancer efficacy is increased by ABTL0812. Due to the unique pharmacological effect, the monotherapy or combination therapy of ABTL0812 presents an acceptable efficacy on high-risk neuroblastoma [179].

#### Z36

According to Yangjie Liao et al., in HeLa cells, the small molecule Z36 promoted autophagy and autophagy death [203]. Analysis of differential gene expression in Z36-treated HeLa cells revealed that Z36-induced ER phagy resulted in ER stress and UPR. It was discovered that Z36 increases the expression levels of LC3, FAM134B and Atg9, which collectively cause excessive enhanced phagocytosis, which is typified by the production of more autophagosomes and larger ones [143].

### Targeting xenophagy

#### Resveratrol

Restorative promotes xenophagy, the autophagy-dependent removal of intracellular microbes in intestinal epithelial cells and macrophages, in a transgenic GFP-LC3 zebrafish model. It’s a particular kind of selective autophagy that breaks down microorganisms inside cells. Increasingly, an increasing number of invasive *Salmonella* and Crohn’s disease-associated adherent-invasive *Escherichia coli* (AIEC) are degraded by xenophagy in resveratrol-induced cells, indicating that resveratrol plays a part in boosting innate immunity. Xenophagy controls intracellular bacteria and alleviates inflammatory response. These results suggest that autophagy-induced nutritional stimulation and/or autophagy restoration may be used to prevent immunological and infectious disorders linked to abnormalities in autophagy [184]. Yuqin Hao et al. discovered that resveratrol induced apoptosis of tumor cells in Squamous cell carcinoma (SCC) by up-regulating p53 protein and mRNA expression and down-regulating SVV protein and mRNA expression [185]. Chang Chao-Hsiang et al. investigated the mechanism of action of resveratrol’s oral anticancer effect on human oral cancer CAR cells that are resistant to cisplatin. Research has demonstrated that resveratrol can regulate autophagy and pro-apoptotic signals, amplify AMPK phosphorylation, and boost the expression of autophagy mRNA genes in CAR cells, including as Atg5, Atg12, Beclin-1, and LC3-II. These findings imply that resveratrol may cause drug-resistant oral cancer cells to undergo autophagy and apoptosis, and that in the near future, which could eventually result in the creation of a novel treatment approach for the illness [186].

#### Tigecycline

Xenophagy is a process to degrade intracellular bacteria, and bacteria infection is a major cause of cancer development. As a result, antibacterial agents may have anti-cancer potentials by inducing xenophagy [148]. The antibiotic drug tigecycline can inhibit gastric cancer cell proliferation by inducing xenophagy but not apoptosis, suggesting that tigecycline may be a candidate for the treatment of gastric cancer patients in preclinical evaluation [148, 204]. Tang et al. revealed that tigecycline can prevent the growth and proliferation of gastric cancer cells by activating and phosphorylating AMPK, and further inhibiting the phosphorylation of mTOR/p70S6K [204]. Their results offer references for tigecycline’s application in the management of gastric cancer. Jakub Rok et al. found that tigacycline can increase the level of autophagy marker LC3A/B protein and effectively inhibit the proliferation of melanoma cells [187].

#### Salinomycin

Salinomycin is a polyether antibiotic agent. Salinomycin is a highly selective potassium ionophore that exhibits anti-cancer properties against various types of cancer cells. In the osteosarcoma cell line U2OS, salinomycin induces apoptosis and xenophagy by generating ROS. Although the role of salinomycin-induced xenophagy in cancer cells remains controversial, it may be a potential anti-cancer strategy via mediating ROS [188].

### Targeting lipophagy

#### Tripterine

Extracted from Tripterygium wilfordii, tripterine is a plant triterpene with possible anti-cancer properties [205, 206]. It triggers lipophagy in human clear cell renal cell carcinoma (ccRCC) cell lines 786-O, A498, SN12C and OS-RC-2 by activating LXRα (liver-X receptors α). Tripterine also promotes cholesterol efflux mediated by ATP-binding cassette transporter A1 (ABCA1), inhibits EMT, and eventually suppresses cancer cell proliferation, migration and invasion. Tripterine may have anti-cancer properties through modulating lipid metabolism and lipophagy [189].

#### PFK158

Ovarian cancer and cervical cancers are two major gynecological cancers that are prone to chemotherapy resistance. A key element in the development of medication resistance is metabolic changes in the lipid and glycolysis pathways [207]. By targeting 6-Phosphofructo-2-Kinase/Fructose-2,6-Biphosphatase 3 (PFKFB3), the new glycolytic inhibitor PFK158 increases lipophagy and boosts chemotherapy sensitivity in gynecologic malignancies. PFK158-induced lipid droplet inhibition involves an increased autophagic flux triggered by p62/SQSTM1 downregulation, increased LC3BII lipidation levels and cytosolic phospholipase A2 (p-cPLA2) downregulation. It is indicated that PFK158 simultaneously targets glycolysis and lipogenesis pathways and promotes lipophagy to inhibit gynecologic cancer growth, which has great clinical significances for overcoming chemotherapy resistance and prolonging the survival of cancer patients [190].

### Targeting lysophagy

#### Loperamide and pimozide

The induction of autophagy holds importance in the management of glioblastoma. Lysophagy and lipotoxicity driven by loperamide and pimozide synergistically induce LMP and cell death by inhibiting the activity of sphingomyelin phosphodiesterase 1 (SMPD1)/ASM and promoting the release of cathepsin B (CTSB) into the cytoplasm of the wild-type MZ-54 cells. Atg5 and Atg7 knockout (KO) cells exhibit drastically reduced LMP and cell death, both of which are enhanced by depletion of valosin containing protein (VCP). These results validated the importance of lysophagy in promoting cell survival, and a promising strategy for treating GBM is to simultaneously induce LMP and hyperactivate autophagy [191].

#### GNS561

GNS561 exerts a high liver tropism and effective anti-cancer activity in the glioblastoma cell line LN-18 and two liver cancer cell line. It is a novel lysophagy inhibitor, and the anti-cancer potential is linked with lysosomal cell death. Due to the lysosomal properties of GNS561, it can reach the enzymatic target palmitoyl-protein thioesterase 1 (PPT1) and inhibit it, resulting in the accumulation of unbound Zn^2+^ in lysosomes, impaired cathepsin activity, blocked autophagic flux, altered mTOR localization, LMP, caspase activation, and cell death [192].

## Summary and prospect

At present, studies have shown that targeted general autophagy is not sufficient for cancer treatment, with risks and certain limitations, while targeted selective autophagy is considered to be a more effective treatment approach [208, 209]. Numerous human diseases have been linked to the pathophysiology of genes related to selective autophagy pathways. Among these, selective autophagy has a dual function in the onset and advancement of cancer and has developed a complex interaction with the proliferation, survival, and progression of cancer cells. In response to intracellular and extracellular stimuli, selective autophagy helps promote cancer development. Conversely, induction of selective autophagy also inhibited the development of cancer. Various types of selective autophagy, including mitophagy, ER-phagy and lysophagy, may provide potential therapeutic targets for cancer therapy. Some selective autophagy studies such as aggrephagy, ferritinophagy, virophagy, etc., are still relatively few, but these types of autophagy have been demonstrated to be connected to the development of cancer [210–212]. This remarkable diversity not only enriches our understanding of the mechanisms of selective autophagy, but also creates an unprecedented opportunity to develop targeted and effective therapeutic interventions in the cancer context. Thus, it is crucial to have a better knowledge of selective autophagy and how it contributes to cancer.

At present, tumor immunotherapy is considered as a promising strategy for cancer treatment [213]. Autophagy controls the immune response by regulating the function of immune cells and the production of cytokines, while cytokines and immune cells can also affect the function of autophagy. For example, the nuclear protein of influenza A virus inhibits innate immune responses by inducing mitophagy [214], and mitophagy-related genes can assess immune activity in pancreatic cancer patients [215]. Many studies have shown that the optimal combination of autophagy-based inducers or inhibitors with multiple therapeutic strategies (including chemotherapy, radiotherapy, immunotherapy, and gene therapy) may be a more effective way to induce tumor cell death [213, 216]. A few autophagy inhibitors have been used in preclinical studies to enhance the anti-tumor effect of immunotherapy. For example, autophagy inhibitor chloroquine can enhance HDIL-2-mediated anti-tumor immunity by enhancing DC, T cells and NK cells, and 3-methyladenine (3-MA) can enhance IL-24-induced apoptosis against oral squamous cell carcinoma. However, induction of autophagy may also favor tumor cells to evade immune surveillance and lead to intrinsic resistance to anti-tumor immunotherapy [217]. Therefore, it remains to be explored whether we should try to enhance or inhibit autophagy in anti-tumor immunotherapy. In the future, in tumor immunotherapy, attention should be paid to how to regulate selected autophagy to strengthen innate and adaptive immune responses and overcome anti-tumor immune resistance.

DNA damage is closely related to the occurrence and development of tumors [183, 218, 219]. When DNA damage occurs, a series of damage response responses are triggered to aid cell survival, including the induction of autophagy. A variety of effectors involved in DNA damage repair, such as ATM, P53 and PARP1, initiate selective autophagy and non-selective autophagy by affecting AMPK, mTOR and some apoptotic proteins [220]. As a degradation pathway, autophagy can directly affect homologous recombination repair, non-homologous end joining repair, and nucleotide excision repair by regulating the level of DNA repair-related proteins to promote DNA repair, and indirectly promote DNA repair by maintaining cellular homeostasis, thus playing an important role in the malignant transformation of normal cells and tumor drug resistance. In addition, autophagy can also be used as a way of programmed cell death when DNA repair fails. Therefore, studying the effect of selective autophagy on tumors by regulating DNA damage repair is of great significance for understanding the mechanism of tumorigenesis and providing treatment ideas [221–223].

Currently available selective autophagy modulators have shown poor bioavailability due to disadvantages such as low solubility in aqueous media, untargeted delivery, toxicity, and resistance associated with higher drug doses, and their use in clinical Settings is limited [224]. Nanotechnology based drug delivery systems show great promise in overcoming these obstacles due to their utilization of the superior drug delivery capabilities of nanocapsules and facilitation of tumor-targeted drug delivery [225]. Co-delivery of selective autophagy modulators and nanocarrier therapeutic agents may result in synergistic therapeutic effects by simultaneously regulating selective autophagy and improving the efficiency of drug delivery. For example, albumin-bound rapamycin can bind hydrophobic drugs to albumin, so it does not need to use toxic solvents, and its anticancer effect can be improved by combining with autophagy inducers. Albumin-bound paclitaxel is a drug approved by FDA for the treatment of advanced breast cancer [226]. Its nanocarrier can deliver the drug to the cancer tissue quickly and stay for a longer time. Some studies have reported that NPs can act as autophagy inducers or autophagy inhibitors [227]. Of course, there is still a long way to go from the design of nanocarriers to clinical applications [228]. Due to the incomplete therapeutic effect of nanoparticles and the off-target toxicity of important organs, the clinical translation of nanoparticles is limited. Further intensified basic research and clinical translation are needed to realize the full potential of in vitro nanoparticle delivery systems.

Targeted selective autophagy anticancer drugs currently in clinical or preclinical trials include natural products and derivatives such as resveratrol, tripterine, small molecule compounds such as ABTL0812, combination drugs such as C150 and gemcitabine for pancreatic cancer, and drug function retargeting such as brigatinib. Despite advances in our understanding of selective autophagy, the translation of mechanistic studies into clinically active drugs remains challenging due to the low absorption and bioavailability of current drugs targeting selective autophagy, which is a highly complex process [229]. Translating biochemical inhibition into comprehensive and selective blockade of selective autophagy pathways in cancer cells is by no means straightforward, thus translating early lead compounds into clinical candidates is more difficult. Progress in the preclinical and clinical development of selective autophagy modulators has been greatly hampered by the lack of selective pharmacological reagents and biomarkers to profile the precise effects of compounds on various forms of autophagy and cellular responses. We must take advantage of modern assays to improve future methods for discovering and validating selective autophagy drugs. To explore and think about natural products and derivatives, small molecule compounds, complexes, combination drugs and functional reorientation of drugs, and further develop more selective and effective anticancer drugs targeting selective autophagy.

In summary, although current studies have shown that selective autophagy plays a dual function in the occurrence and development of cancer, the specific mechanism of selective autophagy and tumorigenesis and development remains to be studied and clarified. In this paper, we reviewed the molecular mechanisms of selective autophagy, introduced the cancer-promoting and cancer-inhibiting effects of selective autophagy in several cancers, and reviewed the tumor therapeutic compounds targeting selective autophagy, aiming to serve as a foundation for the creation of novel biomarkers of selective autophagy levels in tumors and the creation of anti-cancer drugs targeting selective autophagy. To better understand the role of selective autophagy in tumor genesis and development, future research should continue to examine the function of cargo receptors and selective autophagy, the molecular mechanism of tumor cells to open a high level of selective autophagy, and the intricacy of tumor-microenvironment interaction. Through the development of anti-cancer drugs targeting selective autophagy through multi-technology and multi-approach, more in vitro and in vivo experiments and clinical studies can be carried out, so as to better target selective autophagy to improve the clinical outcome of tumor patients.

## Data Availability

No datasets were generated or analysed during the current study.

## Abbreviations

ABCA1: ATP-binding cassette transporter A1
ACBP: acyl-CoA binding protein
ACD: autophagic cell death
AIEC: adherent-invasive *Escherichia coli*
AIM: Atg8-interacting motif
ALK: anaplastic lymphoma kinase
AMACR: Alpha-methylacyl-CoA racemase
ATG: Autophagy-related gene
Atg16L1: autophagy-related 16-like 1
Atg8/LC3/GABARAP: Atg8 family LC3/GABARAP proteins
ATGL: adipose triglyceride lipase
ATL3: Atlastin GTPase 3
ATP: Adenosine triphosphate
BH3: Bcl-2 homology 3
BNIP3: BCL2/adenovirus E1B 19KDa-interacting protein 3
C53: CDK5 regulatory subunit-associated protein 3
CagA: cytotoxin-associated gene A
CALCOCO1: Calcium-binding and coiled-coil domain-containing protein 1
CAMK2B: calcium/calmodulin dependent protein kinase II beta
CCCP: carbonyl cyanide m-chlorophenyl hydrazone
CCPG1: Cell-cycle progression gene 1
ccRCC: clear cell renal cell carcinoma
CK2: casein kinase 2
CQ: chloroquine
CRC: Colorectal cancer
CSCs: cancer stem cells
Ctl: cytotoxic T cells
CTSB: cathepsin B
DDRGK1: DDRGK domain containing 1
DG: diacylglycerol
Drp1: dynein-related protein 1
dsDNA: double-stranded DNA
DTT: dithiothreitol
EMT-TF: epithelial mesenchymal transition
Epr1: ER-phagy receptor 1
ER: Endoplasmic reticulum
ERAD: ER-associated degradation
ESCRT: endosomal sorting complexes needed for transport
FAM134B: Family with sequence similarity 134 members
FAO: fatty acid β-oxidation
FDA: Food and Drug Administration
FIRs: FIP200-interacting regions
FL3: Flavagline
FUNDC1: FUN14 domain containing 1
GABARAP: Gamma-aminobutyric acid receptor-associated protein
Gal: galectin
HCQ: hydroxychloroquine
HECT: homolog of the E6-AP carboxyl terminus
HIF1A: Hypoxia-inducible factor 1-alpha
HK2: hexokinase 2
HSL: Hormone-sensitive lipase
HSS: Hallermann-Streiff syndrome
IDR: intrinsically disordered region
IMM: Inner mitochondrial membrane
KIR: keap1 interaction region
KO: knockout
LAL: lysosomal acid lipase
LDs: lipid droplets
LIR: LC3-interacting region
LMP: Lysosomal membrane permeabilization
LOP: Loperamide
LRSAM1: Leucine-rich repeat and sterile alpha motif-containing protein 1
LVV: LIR motif
3-MA: 3-methyladenine
MARCH5: membrane-associated ring-CH-type finger 5
MG: monoacylglycerol
MGL: Monoacylglycerol lipase
MSDCs: myeloid-derived suppressor cells
MYOF: Myoferritin
NBR1: Neighbor of BRCA1 gene 1
NES: export motif
NIX: BNIP3-like
NLS: nuclear localization signal
NPs: Nanoparticles
Nrf2: Nuclear factor erythroid 2-related factor 2
OA: Oroxylin A
OMM: Outer mitochondrial membrane
ORP8: Oxysterol-binding protein-related protein 8
OSBP: oxysterol binding protein
OXPHOS: Oxidative phosphorylation
p62/SQSTM1: Sequestosome 1
PARK: Parkinson disease protein
*P. pastoris*: *Pichia pastoris*
PB1: Phox-BEM1
PCBP1: poly (rC)-binding protein 1
p-cPLA2: phospholipase A2
PDAC: Pancreatic ductal adenocarcinoma
PEX: peroxin
PFKFB3: 6-Phosphofructo-2-Kinase/Fructose-2,6-Biphosphatase 3
PGAM5: PGAM family member 5
*PgdA*: *peptidoglycan deacetylase*
PHB2: protein called prohibitin 2
PINK1: PTEN-induced kinase 1
PKM2: Pyruvate kinase M2
PLAA: phospholipase A 2-activating protein
PMP70: 70-kDa peroxisomal membrane protein
pnER: perinuclear ER
PPARs: peroxisome proliferator-activated receptors
PPT1: palmitoyl-protein thioesterase 1
*H. Pylori*: *Helicobacter pylori*
RHD: reticular homeodomain
RNS: reactive nitrogen species
ROS: Reactive oxygen species
RPC: pexophagic receptor-protein complex
RTN3L: Reticulon-3 L
(SCF)^FBXO27^: SKP1/CUL1/F-box protein
SCC: Squamous cell carcinoma
SEC62: SEC62 homolog, preprotein translocation factor
Sirna: small interfering rna
*S. cerevisiae*: *Saccharomyces cerevisiae S. cerevisiae*
SMURF1: Smad ubiquitin regulatory factor 1
TAG: Triacylglycerol
TEX264: testis-expressed protein 264
TFEB: Transcription factor EB
TG: Triglycerides
Ub: ubiquitin
UBA: Ubiquitin-associated domain
UBE2QL1: ubiquitin conjugating enzyme E2Q family-like 1
UFL1: UFM1 specific ligase 1
UIR: UDS interface region
ULK1: Unc-51 like autophagy activating kinase 1
UPR: Unfolded protein response
USP: Ubiquitin-specific peptidase
VacA: Cytotoxin A
VCP: valosin containing protein
